# Mito-Nuclear Communication by Mitochondrial Metabolites and Its Regulation by B-Vitamins

**DOI:** 10.3389/fphys.2019.00078

**Published:** 2019-02-12

**Authors:** Joëlle J. E. Janssen, Sander Grefte, Jaap Keijer, Vincent C. J. de Boer

**Affiliations:** Human and Animal Physiology, Wageningen University & Research, Wageningen, Netherlands

**Keywords:** B-vitamins, mito-nuclear signaling, TCA cycle metabolites, acyl-CoA, reactive oxygen species, sirtuins, epigenetic modifications

## Abstract

Mitochondria are cellular organelles that control metabolic homeostasis and ATP generation, but also play an important role in other processes, like cell death decisions and immune signaling. Mitochondria produce a diverse array of metabolites that act in the mitochondria itself, but also function as signaling molecules to other parts of the cell. Communication of mitochondria with the nucleus by metabolites that are produced by the mitochondria provides the cells with a dynamic regulatory system that is able to respond to changing metabolic conditions. Dysregulation of the interplay between mitochondrial metabolites and the nucleus has been shown to play a role in disease etiology, such as cancer and type II diabetes. Multiple recent studies emphasize the crucial role of nutritional cofactors in regulating these metabolic networks. Since B-vitamins directly regulate mitochondrial metabolism, understanding the role of B-vitamins in mito-nuclear communication is relevant for therapeutic applications and optimal dietary lifestyle. In this review, we will highlight emerging concepts in mito-nuclear communication and will describe the role of B-vitamins in mitochondrial metabolite-mediated nuclear signaling.

## Introduction

**FIGURE 1:**
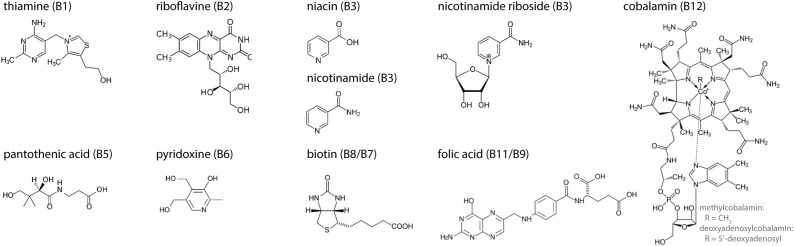
Molecular structures of the eight B-vitamins. Three forms are given for vitamin B3: niacin, nicotinamide, and nicotinamide riboside. R-group in vitamin B12 represents a methyl-group (methylcobalamin) or a 5′-deoxyadenosyl-group (deoxyadenosylcobalamin).

**FIGURE 2:**
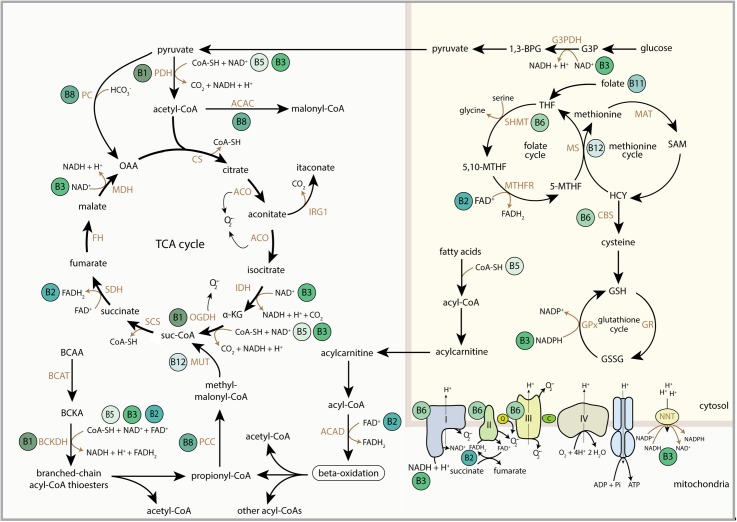
Schematic overview of the regulatory role of B-vitamins in mitochondrial and cytosolic metabolic reactions. FAD, flavin adenine dinucleotide; FADH_2_, hydroquinone form of FAD; NAD^+^, nicotinamide adenine dinucleotide; NADH, reduced form of NAD^+^; CoA-SH, Coenzyme A; PDH, pyruvate dehydrogenase complex; ACAC, acetyl-CoA carboxylase; CS, citrate synthase; ACO, aconitase; IRG1, immunoresponsive gene 1; IDH, isocitrate dehydrogenase; α-KG, alpha-ketoglutarate; OGDH, 2-oxoglutarate dehydrogenase complex; suc-CoA, succinyl-CoA; SCS, succinyl coenzyme A synthetase; SDH, succinate dehydrogenase; FH, fumarate hydratase; MDH, malate dehydrogenase; OAA, oxaloacetate; BCAA, branched-chain amino acids; BCAT, branched-chain amino acid transaminase; BCKA, branched-chain ketoacids; BCKDH, branched-chain ketoacid dehydrogenase; PCC, propionyl-CoA carboxylase; MUT, methylmalonyl-CoA mutase; ACAD, acyl-CoA dehydrogenase; 1,3-BPG, 1,3-bisphosphoglyceric acid; G3PDH, glyceraldehyde 3-phosphate dehydrogenase; G3P, glyceraldehyde 3-phosphate; THF, tetrahydrofolate; SHMT, serine hydroxymethyltransferase; 5,10-MTHF, 5,10-methylenetetrahydrofolate; MTHFR, methylene tetrahydrofolate reductase; 5-MTHF, 5-methyltetrahydrofolate; MS, methionine synthase; MAT, methionine adenosyltransferase; SAM, *S*-adenosylmethionine; HCY, homocysteine; CBS, cystathionine-beta-synthase; GSH, glutathione; GSSG, glutathione disulphide; GPx, glutathione peroxidase; GR, glutathione reductase; Q, coenzyme Q; C, cytochrome C; NNT, nicotinamide nucleotide transhydrogenase; OMM, outer mitochondrial membrane; IMM, inner mitochondrial membrane.

B-vitamins are water-soluble vitamins (Figure 1) that are essential nutrients in supporting mitochondrial function, predominantly by serving as nutritional cofactors or coenzymes for enzymes that are located in mitochondria (Figure 2 and Table 1) (Depeint et al., 2006a,b; Keijer et al., 2011). Five out of the eight B-vitamins are directly involved in functioning of the tricarboxylic acid (TCA) cycle (B1, B2, B3, B5, and B8/B7) (Figure 2). Vitamin B6 is required for iron-sulfur (FeS) biosynthesis, *de novo* synthesis of NAD^+^ and substrate metabolism, whereas vitamin B11/B9 and B12 are essential in nucleotide biosynthesis and amino acid metabolism (Figure 2). Vitamin B12 is also crucial for the generation of succinyl-CoA from methylmalonyl-CoA in the mitochondria (Figure 2). Since the activity of mitochondrial enzymes is regulated by B-vitamin levels, maintaining a balanced pool of B-vitamins in the mitochondria is essential to support the metabolic and other biochemical reactions that are orchestrated by these mitochondrial enzymes.

**Table 1:** B-vitamins and their generic names, bioactive forms, recommended daily intake values and rich dietary sources.

Vitamin	Generic name	Bioactive form	PRI/AI^1,2^	Rich dietary sources^1^
B1	Thiamin	Thiamin diphosphate	PRI 0.1 mg/MJ/day	Whole grains, legumes, meat, liver, and fish.
B2	Riboflavin	FMN, FAD	PRI 1.6 mg/day	Milk, milk products, eggs.
B3	Niacin (nicotinic acid, nicotinamide, nicotinamide riboside, nicotinamide mononucleotide)	NAD^+^, NADP^+^	PRI 1.6 mg NE^3^/MJ/day	Liver, meat, and meat products, fish, peanuts, coffee and whole grains. Also in protein-rich foods, such as milk, cheese and eggs (good source of tryptophan = 60 NE).
B5	Pantothenate	Coenzyme A (CoA)	AI 5 mg/day	Meat products, bread, milk-based products, eggs, nuts, and vegetables.
B6	Pyridoxal, pyridoxol or pyridoxamine	Pyridoxal phospate	PRI 1.6–1.7 mg/day	Grains, legumes, nuts, seeds, potatoes, some herbs and spices, meat and meat products, fish.
B8/B7	Biotin	Biotin	AI 40 μg/day	Especially in liver and eggs, but also in mushrooms and some cheeses. Lower amounts in lean meat, fruit, cereals, and bread.
B11/B9	Folate	MTHF, BH4, and others	PRI 330 μg DFE^4^/day	Dark green leafy vegetables, legumes, orange, grapefruit, peanuts and almonds, but also in liver, kidney, and yeast.
B12	Cobalamin	Deoxyadenosylcobalamin, Methylcobalamin	AI 4 μg/day	Meat, liver, milk, and eggs.


In order to maintain a balanced B-vitamin pool, dietary consumption of foods rich in B-vitamins is necessary, as B-vitamins cannot be synthesized by the body and must be derived from the diet. The European Food and Safety Authority (EFSA) has established the daily intake requirement for each B-vitamin (Table 1). Daily intake requirements are expressed as population reference intake (PRI) or adequate intake (AI) and are dependent on population group, age and/or gender. To meet these requirements, it is recommended to have a daily consumption of dietary sources that are rich in B-vitamins (Table 1). Some of these food products contain only one of the B-vitamins, whereas others provide several B-vitamins. For example, dark leafy green vegetables are rich in vitamin B11, whereas eggs contain vitamin B2, B5, B8 as well as B12. In general, a diverse diet will meet the recommended daily intake requirements, but insufficient intake of a food group that exclusively provides a specific B-vitamin requires alternative dietary adjustments. As an example, vitamin B12 is mainly provided by animal sources, especially meat, and cannot be derived from plant sources. Individuals who do not consume meat products, such as vegetarian or veganists, should consider the consumption of alternative foods that are fortified with vitamin B12 or should perhaps take supplementation with vitamin B12.

Sufficient B-vitamin intake is essential to maintain mitochondrial function, control levels of mitochondrial metabolites and prevent disease. Evidence is emerging that the wide array of metabolites that are produced in the mitochondria do not only support mitochondrial respiration and ATP generation, but can also communicate with other parts of the cell, including the nucleus (Quiros et al., 2016). This retrograde signaling from mitochondria to the nucleus is called mito-nuclear communication and allows mitochondria to regulate multiple cellular processes, including cell cycle decisions, cell signaling and epigenetic regulation (Weinberg et al., 2015; Vyas et al., 2016). Dysregulation of the interplay between mitochondrial metabolites and the nucleus has been established to play a direct role in aging and several disease pathologies (Nunnari and Suomalainen, 2012), including cancer (Vyas et al., 2016; Cannino et al., 2018), inflammation (West et al., 2011) and ischemia/reperfusion (I/R) events (Murphy and Hartley, 2018). Although B-vitamins have direct effects on mitochondrial function, the role of B-vitamins in mito-nuclear communication has been poorly described. Understanding the role of B-vitamins can be relevant for designing novel therapeutic applications or developing new studies that focus on dietary lifestyle changes. Here, we will outline the role of B-vitamins in mitochondrial function, highlight the emerging concepts in the communication between mitochondrial metabolites and the nucleus and identify how B-vitamins can regulate mito-nuclear communication.

## Regulation of Mitochondrial Metabolism by B-Vitamins

### Vitamin B1 – Thiamine-Diphosphate

Vitamin B1 (thiamine) is highly enriched within the mitochondria, as they contain more than 90% of all cellular thiamine (∼30 μM) (Bettendorff et al., 1994). The active form of thiamine, thiamine-diphosphate, is an essential cofactor of multiple mitochondrial dehydrogenase complexes, including the pyruvate dehydrogenase (PDH) complex, the alpha-ketoglutarate (α-KG) dehydrogenase (OGDH) complex and branched-chain keto-acid dehydrogenase complex (BCKDH) (Figure 2). Recent analyses also showed that thiamine and its derivatives can allosterically regulate malate dehydrogenase and glutamate dehydrogenase, both involved in the malate-aspartate shuttle, thereby decreasing the efflux of citrate from the mitochondria and increasing citrate flux through the TCA cycle (Mkrtchyan et al., 2015).

### Vitamin B2 – FAD and FMN

Vitamin B2 (riboflavin) exists in two bioactive forms, flavin adenine dinucleotide (FAD) and flavin mononucleotide (FMN). Riboflavin is first phosphorylated by riboflavin kinase, generating FMN, which can be further converted into FAD by FAD synthase (FADS) that subsequently transfers an AMP unit from ATP to FMN. By acting as electron carriers, FAD and FMN comprise the essential prosthetic groups in flavoproteins. About 90 flavoproteins are identified in humans (Macheroux et al., 2011), the majority harboring FAD. They are mainly located in the mitochondria and catalyze a variety of redox reactions, including oxidation, reduction and dehydrogenase reactions (Figure 2). For example, mitochondrial acyl-CoA dehydrogenases, which perform the first step in fatty acid beta-oxidation, compromise a large group of FAD-dependent flavoproteins. Riboflavin also supports the redox reactions catalyzed by succinate dehydrogenase (SDH) and glutathione reductase (GR) by supplying FAD. Reduction of FAD to FADH_2_ is an intermediary step in the oxidation of succinate to fumarate by SDH. Reduction of oxidized glutathione (GSSG) to 2 molecules of glutathione (GSH) by FAD-dependent GR utilizes the reduction of FAD to FADH_2_ as an intermediate step, which is also coupled to the reduction of NADPH to NADP^+^. In this way, riboflavin supports anti-oxidant defense mechanisms by serving GSH metabolism (Ashoori and Saedisomeolia, 2014), but riboflavin is also proposed to act as an anti-oxidant by its own oxidation (Toyosaki, 1992). Riboflavin also supports NADPH-dependent biliverdin reductase B (BLVRB) (Hultquist et al., 1993), which is involved in protection against I/R oxidative injuries (Ashoori and Saedisomeolia, 2014; Sanches et al., 2014).

### Vitamin B3 – NAD^+^ and NADP^+^

Vitamin B3 is also referred to as niacin, which comprises the various dietary forms of vitamin B3, nicotinic acid (NA), nicotinamide (NAM), as well as the recently recognized nicotinamide riboside (NR), and nicotinamide mononucleotide (NMN) (Yoshino et al., 2018). These forms have different bioactivation routes toward NAD^+^. In addition, NAD^+^ can also be synthesized *de novo* from the essential amino acid tryptophan in a ratio of approximately one to 60, meaning that sixty times as much milligrams of tryptophan is needed to generate each gram of NAD^+^, than is generated from each milligram of vitamin B3. As a coenzyme, NAD^+^ is principally used as an electron acceptor (Figure 2) (Xiao et al., 2018). Furthermore, NAD^+^ can be converted in a second, distinct cofactor form, NADP^+^, with a principal role in lipid metabolism and redox homeostasis. NAD^+^ is reduced to NADH by two electrons that are donated mostly by catabolic intermediates in mitochondrial substrate oxidation, especially in the TCA cycle. NADH is primarily used to feed electrons to the electron transport system (ETS) and to provide reduction equivalents to regenerate redox systems, including NADPH. Nicotinamide nucleotide transhydrogenase (NNT) catalyzes NADPH generation from NADH in a proton gradient dependent manner (Murphy, 2015). NADPH can also be generated from other sources, including the pentose phosphate pathway, the serine synthesis pathway and glutamate dehydrogenase (Lewis et al., 2014; Ronchi et al., 2016).

Apart from mediating mitochondrial metabolic signals via the electron carrier properties, especially NAD^+^ is also a direct regulator of protein post-translational acylation and ADP-ribosylation modifications (Houtkooper et al., 2010). NAD^+^ is a co-substrate for three protein modifying enzyme families; the NAD-dependent deacylases (sirtuins, SIRT), poly-ADP ribosylation polymerases (PARPs) and mono-ADP ribosyltransferases (ARTs) (Fang et al., 2017; Ryu et al., 2018). For each protein modification catalyzed by these enzymes, one NAD^+^ molecule is consumed. Recent studies demonstrated that these reactions account for the use of two-thirds of the total cellular pool of NAD^+^ (Liu et al., 2018), highlighting the importance of NAD^+^ as precursor for protein modifications in the cell.

### Vitamin B5 – Coenzyme A

Vitamin B5 (pantothenate) is the precursor for biosynthesis of Coenzyme A (CoA). Lipmann et al. (1947) were the first to describe CoA as a coenzyme that transfers acyl groups and functions as a carrier of acyl moieties (Lipmann et al., 1947). In addition to its role in acyl transferase reactions (Figure 2) (Pietrocola et al., 2015; Sabari et al., 2017), CoA is the balancing factor between carbohydrate and lipid metabolism during glucose oxidation in the TCA cycle versus fatty acid oxidation (Leonardi et al., 2005), and it is a required cofactor for the biosynthesis of ketone bodies (McGarry and Foster, 1980; Puchalska and Crawford, 2017). These metabolic functions explain why CoA is predominantly present in the mitochondria (2.2 mM), with less occurrence in the peroxisomes (20–140 μM) and to some extent in the cytoplasm (less than 15 μM) (Williamson and Corkey, 1979).

### Vitamin B6 – Pyridoxal Phosphate

The active form of vitamin B6 (pyridoxal phosphate) is generated by distinct modification pathways that depend on the form of vitamin B6 (pyridoxal, pyridoxol, or pyridoxamine) that is available. Pyridoxal phosphate plays a major role in energy metabolism, but is particularly involved in amino acid metabolism, *de novo* NAD^+^ and FeS biosynthesis, and by functioning as a cofactor for several aminotransferases and decarboxylases (Figure 2) (Depeint et al., 2006a; Braymer and Lill, 2017). FeS clusters are integral parts of many metabolic protein complexes, such as aconitase and ETS complexes, as well as other cellular protein complexes, such as DNA polymerases and helicases (Braymer and Lill, 2017). Furthermore, pyridoxal phosphate is the essential coenzyme for mitochondrial aminolevulinate synthase, which is essential for synthesis of heme (Scholnick et al., 1972).

### Vitamin B8/B7 – Biotin

Vitamin B8/B7 (biotin) is used in organisms without further chemical or enzymatic modification. The cellular localization of biotin is consistent with its function, with enriched fractions in the mitochondria and cytosol (Petrelli et al., 1979). Biotin acts as an essential coenzyme for five carboxylases from which four are located within the mitochondria (Figure 2). These carboxylases are carboxyl transferases that catalyze the addition of a carboxylic acid group to an organic compound, a reaction that utilizes CO_2_. Pyruvate carboxylase (PC) converts pyruvate into oxaloacetate (OAA) and functions to resupply the TCA cycle, but also in the initial step of gluconeogenesis (in liver and kidney) and lipogenesis (in adipose tissue, liver, brain). Propionyl-CoA carboxylase (PCC) converts propionyl-CoA to methylmalonyl-CoA with a key role in the catabolism of amino acids (isoleucine, valine, methionine, and threonine) and odd-chain fatty acids. Methylcrotonyl-CoA carboxylase (MCCC) converts 3-methylcrotonyl-CoA to 3-methylglutaconyl-CoA, thus having a critical step in leucine and isovaleric acid catabolism. Acetyl-CoA carboxylase B (ACACB) is a biotin carboxyl carrier protein and can function as a biotin carboxylase and carboxyltransferase. As carboxyltransferase, it catalyzes the ATP-dependent carboxylation of acetyl-CoA to malonyl-CoA. It is localized in the mitochondrial outer membrane and associates with carnitine palmitoyltransferase 1 (CPT1) allowing it to perform its decisive role in channeling acetyl-CoA toward either lipid synthesis in the cytosol or mitochondrial beta-oxidation. Cytoplasmic biotin containing acetyl-CoA carboxylase (ACACA) has similar metabolic functions and has a key role in long-chain fatty acid biosynthesis.

### Vitamin B11/B9 – Folate, Methyltetrahydrofolate and Others

Folate is the generic name for various different forms of this vitamin, also being referred to as vitamin B11 or B9. Several key steps of folate metabolism occur in mitochondria, and 30–50% of all cellular folate is located within the mitochondria (Appling, 1991; Tibbetts and Appling, 2010). Similar to vitamin B3 metabolism, folate metabolism is highly complex, especially because of the wide variety of reactions in which folate is involved (Depeint et al., 2006a; Fox and Stover, 2008; Nijhout et al., 2008). Folate in its various cofactor forms is essential for the synthesis of ADP and GDP, synthesis of purines and thymidylate, providing the methylation donor *S*-adenosylmethionine (SAM), for cellular GSH metabolism, and for amino acid metabolism, with methionine recycling occurring in the cytoplasm and serine-glycine interconversion taking place in mitochondria (Figure 2) (Reed et al., 2008; Ulrich et al., 2008). The methionine derivative SAM supports more than 100 transmethylation reactions by acting as a universal methyl donor.

### Vitamin B12 – Deoxyadenosylcobalamin and Methylcobalamin

Vitamin B12 (cobalamin) is structurally the most complex and largest B-vitamin and is required as a coenzyme in both the mitochondria and cytosol. Deoxyadenosylcobalamin is the essential cofactor of methylmalonyl-Coenzyme A mutase (MUT) in mitochondria, which converts methylmalonyl-CoA into succinyl-CoA, and has a role in the degradation of the amino acids and odd-chain fatty acid, just like PCC (Figure 2). Adenosylcobalamin was shown to support the catabolism of branched-chain amino acids (BCAAs) that are utilized for fatty acid synthesis in differentiating adipocytes (Green et al., 2016). Adenosylcobalamin deficiency also resulted in accumulation of methyl-malonic acid (MMA), methylmalonyl-CoA, and odd-chain fatty acids, indicating that cobalamin is crucial for MUT function (Green et al., 2016). Methylcobalamin is the cofactor for cytosolic 5-methyltetrahydrofolate-homocysteine methyltransferase (MTR), also known as methionine synthase (MS), which catalyzes the transmethylation of homocysteine by methyltetrahydrofolate (MTHF) to methionine (Figure 2) (Ludwig and Matthews, 1997; Matthews et al., 1998). The other product of the MS reaction is THF, the fully reduced form of folate, making folate metabolism critically dependent on sufficient availability of methylcobalamin (Scott, 1999). Furthermore, by acting as coenzyme for MS, methylcobalamin contributes to the synthesis of GSH (Pastore et al., 2014).

## Regulation of Mito-Nuclear Communication by B-Vitamins

In the different mitochondrial reactions that are supported by B-vitamins, a diverse array of metabolites are generated. These metabolites are not only drivers of cellular metabolism and respiration, they can also forward metabolic signals to the nucleus. In this way, they maintain metabolic homeostasis, but also facilitate the cell to dynamically respond to environmental stress signals, like nutrient deprivation and oxidative stress (Quiros et al., 2016). Mitochondrial metabolites that perform key signaling roles in mito-nuclear communication are generated in the TCA cycle, in fatty acid and amino acid oxidation pathways, as well as in the ETS. Their signaling roles center mainly around (1) regulating cytosolic and nuclear dioxygenases, hydroxylases, and NAD-dependent deacylases, (2) functioning as substrate or precursor for protein post-translational modification (PTM), and (3) acting as electron donor or acceptor for redox reactions. B-vitamins alter the levels of mitochondrial signaling metabolites, and consequently impact their mito-nuclear signaling roles.

Below, we will describe four mito-nuclear communication pathways involving mitochondrial metabolites, and highlight the impact of B-vitamins on these pathways. Firstly, hypoxia-inducible factor 1 (HIF1) signaling is regulated by the TCA cycle intermediates α-KG, succinate and fumarate via activating or inhibiting HIF1-regulating hydroxylases. Second, the same TCA cycle intermediates also mediate the regulation of dioxygenases involved in methylation status of DNA and histones in the nucleus. Third, acyl-CoA molecules coming from TCA cycle, fatty acid oxidation and amino acid metabolism, are substrates for acylation modifications of histones. Fourth, antioxidant and redox signaling pathways are altered by TCA cycle intermediates as well as mitochondrial-derived reactive oxygen species (ROS).

### HIF1 Signaling

**FIGURE 3:**
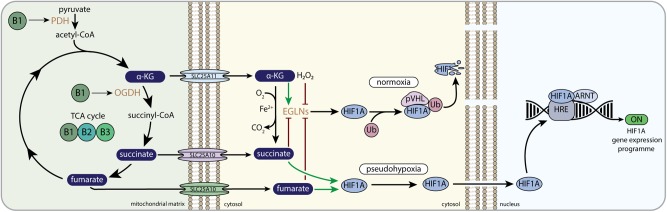
B-vitamins and HIF1-related mito-nuclear signaling. PDH, pyruvate dehydrogenase complex; α-KG, alpha-ketoglutarate; OGDH, 2-oxoglutarate dehydrogenase complex; SLC25A11, mitochondrial 2-oxoglutarate/malate carrier protein; SLC25A10, mitochondrial dicarboxylate carrier; EGLNs, egg-laying-defective nine family or HIF prolyl hydroxylases; Ub, ubiquitin E3 ligase; pVHL, von Hippel–Lindau protein; HIF1A, hypoxia-inducible factor 1 alpha; ARNT, aryl hydrocarbon receptor nuclear translocator; HRE, hypoxia response element; OMM, outer mitochondrial membrane; IMM, inner mitochondrial membrane.

Hypoxia-inducible factor 1 (HIF1) signaling mediates the physiological response to hypoxia. Dimerization of hypoxia-inducible factor 1 alpha (HIF1A) with the aryl hydrocarbon receptor nuclear translocator, ARNT (HIF1B), allows the transcription factor to bind to the hypoxia responsive elements (HRE) in a variety of genes that orchestrate adaptation to hypoxia and restoration of oxygen supply (Schofield and Ratcliffe, 2004) (Figure 3). HIF mediated transcription is dependent on the binding of the co-activators EP300 (E1A binding protein p300) and Creb binding protein (CREBBP). Apart from regulating cellular metabolic pathways in response to hypoxia, HIF1 also regulates red blood cell biosynthesis, iron metabolism and the formation of new blood vessels by coordinating the expression of angiogenic growth factors. In this way it plays a crucial role in regulating the response to hypoxia (Semenza, 2017) as well as vascularization of the developing embryo (Macklin et al., 2017). Dysregulation of the HIF1 signaling pathway occurs in multiple pathologies. Cancer cells benefit from altered control of the HIF1 signaling pathway (Schito and Semenza, 2016), HIF1 is often associated with acute or chronic inflammatory disorders (Imtiyaz and Simon, 2010), and HIF1 plays a role in the pathology of insulin resistance as well as non-alcoholic fatty liver disease (Lefere et al., 2016).

The oxygen-sensitive HIF1A subunit is regulated by the Egl-Nine (EGLN, also called PHD) prolyl hydroxylase enzymes. EGLNs are part of a large Fe(II)/α-KG dependent dioxygenase family, that also consists of the ten-eleven translocation (TET) dioxygenases and the Jumonji C-domain-containing histone lysine demethylases (JmJ-KDM). EGLN catalyzes the hydroxylation of prolyl residues on HIF1A, which allows the interaction with von Hippel-Lindau protein (pVHL) that is part of a multimeric protein complex that contains ubiquitin E3 ligase activity. Ubiquitination by the pVHL complex promotes the degradation of HIF1A when normal oxygen levels are present (Figure 3) (Schofield and Ratcliffe, 2004). In low oxygen levels (i.e., hypoxia), HIF1A escapes proteosomal degradation, because EGLN cannot hydroxylate HIF1A, which allows HIF1A to bind to ARNT and translocate to the nucleus. Here, the functional HIF1 complex is fully assembled, thereby promoting the expression of multiple genes involved in cellular survival during hypoxia (Figure 3) (Maxwell et al., 1999; Schofield and Ratcliffe, 2004; Aragones et al., 2009). Assembly of the functional HIF1 complex in the nucleus, i.e., the binding to EP3000 and CREBBP, is also dependent on the absence of HIF asparaginyl hydroxylation by HIF1AN (hypoxia-inducible factor 1, alpha subunit inhibitor; also called FIH). Similar to EGLN, HIF1AN is dependent on oxygen, Fe(II), ascorbate, and α-KG (Bishop and Ratcliffe, 2015).

EGLNs and HIF1AN use the TCA cycle intermediate α-KG as co-substrate to catalyze their Fe(II)-dependent hydroxylation reaction (Figure 3). One oxygen atom of O_2_ is donated to CO_2_ when α-KG is converted to succinate, whereas the other oxygen of O_2_ is used for the hydroxylation of the proline residues (Aragones et al., 2009). The direct involvement of α-KG in the EGLN and HIF1AN reaction connects mitochondrial-derived α-KG directly to HIF1 signaling. Moreover, both succinate and fumarate have been shown to interfere with the α-KG dependent EGLN reaction (Koivunen et al., 2007; Serra-Perez et al., 2010; Rose et al., 2011), whereas citrate was shown to interfere with HIF1AN and EGLN *in vitro* as well (Hewitson et al., 2007; Koivunen et al., 2007). This links multiple TCA cycle metabolites other than α-KG to HIF1 signaling. In normal cell physiology it was shown that α-KG was needed and could be limiting for activation of EGLNs upon reoxygenation after anoxic culturing conditions (Serra-Perez et al., 2010). When cells were cultured in low nutrient conditions and oxygen deprivation, the EGLN co-substrate α-KG was low, whereas the EGLN inhibitor fumarate was normal, indicating that limiting α-KG levels could prevent EGLN activation upon reoxygenation and thus prevent the proteasomal breakdown of HIF1 (Serra-Perez et al., 2010).

Since HIF1 signaling is often found to be upregulated in tumors and mutations in the pVHL gene are causing the hereditary cancer syndrome, von Hippel-Lindau disease, HIF1 signaling is studied extensively in the context of cancer (Schito and Semenza, 2016). The TCA cycle metabolites fumarate and succinate are now known to be able to behave as oncometabolites in cancers. Oncometabolites have been defined as small molecule components of normal metabolism whose accumulation causes cellular dysregulation and consequently primes cells allowing future progression to cancer (Yang et al., 2012). Since oncometabolites are structurally similar to α-KG, they can activate tumorigenic pathways by acting as competitive inhibitors of Fe(II)/α-KG-dependent EGLNs, as well as other dioxygenases, to interfere with HIF1 signaling, finally contributing to tumorigenesis (Gottlieb and Tomlinson, 2005). Many studies have demonstrated that succinate and fumarate promote tumorigenesis by stabilizing HIF1A via EGLN inhibition (Figure 3) (Gimenez-Roqueplo et al., 2001; Briere et al., 2005; Dahia et al., 2005; Isaacs et al., 2005; Pollard et al., 2005; Selak et al., 2005; Koivunen et al., 2007), although succinate was also found to alter EGLN activity via a HIF-independent mechanism (Lee et al., 2005).

In addition to several cancer pathologies, succinate-induced HIF1A stabilization has been demonstrated to play a role in inflammation (Tannahill et al., 2013; Cordes et al., 2016; Lampropoulou et al., 2016) and rheumatoid arthritis (Li Y. et al., 2016, 2018). Activation of macrophages with lipopolysaccharide (LPS) impaired SDH function, thereby boosting the levels of succinate (Tannahill et al., 2013), and inducing HIF1-mediated secretion of pro-inflammatory interleukin (IL)-1beta (Tannahill et al., 2013; Lampropoulou et al., 2016). In an *in vivo* model of rheumatoid arthritis, transforming growth factor beta (TGF-β) induction also resulted in accumulating succinate levels, which were found to activate the NLRP3 inflammasome in a HIF1A-dependent manner (Li Y. et al., 2016). The same authors recently demonstrated that succinate also boosted HIF1A-mediated vascular endothelial growth factor production and angiogenesis (Li Y. et al., 2018).

#### B-Vitamin Regulation of HIF1 Signaling

Multiple B-vitamins maintain mitochondrial function. Therefore, alterations in the TCA cycle intermediates α-KG, succinate and fumarate caused by alterations in B-vitamin levels, likely impact HIF1 signaling through the α-KG-dependent EGLNs. Indeed, dysregulation of mitochondrial NAD^+^ metabolism by knockdown of the nicotinamide nucleotide transhydrogenase (NNT) gene in cells, caused accumulation of α-KG relative to succinate levels, which lowered HIF1A stability and HIF1A target gene expression (Ho et al., 2017). HIF1A could be stabilized again by addition of dimethylsuccinate, a cell permeable form of succinate (Ho et al., 2017). Vitamin B3 supplementation (in the form of NMN) was able to rescue a pseudo-hypoxic state, characterized by HIF1A stability in muscle during normoxia, that was induced by aging in mice (Gomes et al., 2013). NMN supplementation failed to rescue pseudo-hypoxia in EGLN KO mice as well as in SIRT1 KO mice (Gomes et al., 2013), implying either a direct role of NAD^+^ availability on SIRT1 activity and HIF1 signaling or an indirect role via regulation of TCA cycle metabolites. This also shows that alterations in TCA cycle metabolites by NMN supplementation could directly impact EGLN activity and thus HIF1 stability. Furthermore, in a glaucoma mouse model, mitochondrial aberrations and low NAD^+^ were observed in retina with increasing age, which made mice more vulnerable to high intra-ocular pressure which is an important risk factor for glaucoma (Williams et al., 2017). Increasing NAD^+^ levels by administering vitamin B3 (in the form of nicotinamide), prevented the mice from developing glaucoma. Interestingly, levels of HIF1 were observed to be increased in glaucoma mice, and expression of HIF1 was decreased by vitamin B3 administration (Williams et al., 2017).

Nicotinamide has also been shown to have a protective role in I/R events. Nicotinamide administration to rats before an experimentally induced ischemic event in the brain, lowered infarct volume, which was attributed to elevated NAD^+^ levels in specific brain areas (Sadanaga-Akiyoshi et al., 2003). Also, NMN, administered during reperfusion after an ischemic event in mice, reduced hippocampal injury significantly by increasing brain NAD^+^ levels (Park et al., 2016). Although the mechanisms behind the effects of vitamins or NAD^+^ on I/R are not completely clear, both ROS signaling and TCA cycle metabolite signaling to HIF1 could play a role. Activation of HIF1A is generally considered to be protective in I/R, but sustained HIF1A expression could also be detrimental in the long-term (Shoji et al., 2014). Ischemic preconditioning of the heart protects the heart from experimental, otherwise lethal, ischemic events. Mitochondrial ROS generation and stabilization of HIF1A were shown to play a role in the protective effect of ischemic preconditioning (Howell and Tennant, 2014).

Vitamin B2 generates the necessary FAD for complex (C) II (SDH) to function in the mitochondrial ETS. Mutations in SDH can either cause a hereditary form of cancer or results in a genetic mitochondrial respiratory chain defect, clinically characterized by a mitochondrial encephalomyopathy (Van Vranken et al., 2015). In fibroblasts derived from patients with a clinical CII deficiency, due to mutations in a SDH assembly protein (SDH assembly factor 1, SDHAF1), HIF1A expression was increased, likely because of accumulation of succinate, that would competitively inhibit EGLNs (Maio et al., 2016). Interestingly, vitamin B2 supplementation lowered succinate levels, by stabilizing the SDH complex, which concomitantly lowered HIF1A expression (Maio et al., 2016). This is in line with clinical data showing that patients with SDHAF1 mutations responded positively to oral riboflavin therapy (Bugiani et al., 2006), and highlighting that increasing mitochondrial vitamin B2 impacts HIF1 signaling.

Apart from regulating (pseudo)hypoxia by TCA cycle intermediates, also pyruvate and lactate have been shown to induce a pseudo-hypoxic state. Pyruvate has been suggested to bind to EGLNs catalytic site, thereby inhibiting EGLN activity and stabilizing HIF1A (Lu et al., 2005). Since vitamin B1 (thiamine) is essential for enzymatic activity of PDH and OGDH, thiamine deficiency decreases the activities of both PDH and OGDH (Figure 3), which is clinically characterized by increased plasma levels of pyruvate and lactate (Frohman and Day, 1949; Park and Gubler, 1969; Falder et al., 2010; Sweet and Zastre, 2013; Hernandez-Vazquez et al., 2016). The elevated pyruvate levels observed in B1 deficiency could impact HIF1 signaling. Although increased levels of α-KG are expected to induce HIF1A degradation and would thus have opposite effects on HIF1A signaling compared to above described effect, the consequences of increased α-KG levels, as reported in thiamine deficiency (Bettendorff et al., 1995), on α-KG-induced nuclear signaling pathways, have not been studied in detail.

Combined, multiple studies point to a role of B-vitamins in regulating HIF1 signaling, but the mechanisms are not sufficiently understood yet. It is likely that B-vitamins could alter the dynamic interplay between TCA cycle metabolites, ROS as well as other metabolites that have been shown to interfere with EGLN activities, which will result in B-vitamin mediated control over the HIF1 signaling pathway.

### Histone and DNA Methylation Regulation

**FIGURE 4:**
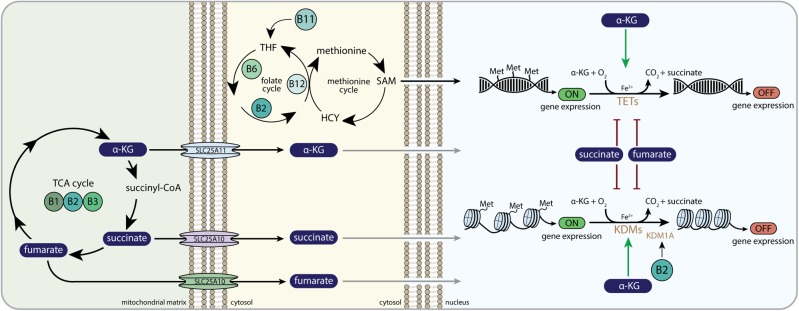
B-vitamins and DNA and histone methylation. α-KG, alpha-ketoglutarate; SLC25A11, mitochondrial 2-oxoglutarate/malate carrier protein; SLC25A10, mitochondrial dicarboxylate carrier; THF, tetrahydrofolate; HCY, homocysteine; SAM, S-adenosylmethionine; Met, methyl-group (CH_3_); TETs, ten-eleven translocation family of DNA demethylases; KDMs, histone lysine demethylase family; KDM1A, histone lysine demethylase family 1A or lysine-specific histone demethylase 1A (LSD1); OMM, outer mitochondrial membrane; IMM, inner mitochondrial membrane.

Apart from regulation of HIF1 signaling by the B-vitamins, other mito-nuclear signaling pathways are also targeted by the B-vitamins. Dioxygenases similar to the EGLN prolyl hydroxylases, regulate demethylation of DNA and histones. The TET dioxygenases catalyze DNA demethylations through 5-methylcytosine (5-mC) hydroxylation (An et al., 2017), and the KDM lysine demethylases catalyze demethylation of histone proteins (Klose et al., 2006). Again, as is the case for EGLN, these oxidative reactions use O_2_ and α-KG to generate CO_2_ and succinate as co-products, the latter acting also as a competitive inhibitor of the α-KG dependent dioxygenases reaction itself (Figure 4) (Hewitson et al., 2007). Thus, α-KG, succinate and fumarate are able to alter the dynamics of DNA and histone methylation through their interaction with TET and KDM demethylation proteins, resulting in the regulation of the epigenetic code and corresponding gene expression programs (Islam et al., 2018). Several studies have demonstrated that reduced α-KG availability drives cancer and stem cell development by lowering dioxygenase activities. For example, reduced levels of α-KG due to BCAA transaminase 1 (BCAT1) overexpression in acute myeloid leukemia (AML) cells were found to inhibit TET demethylase activity, thereby inducing DNA hypermethylation, which promoted AML cell survival and lead to decreased clinical outcome (Raffel et al., 2017). Furthermore, exogenous α-KG supplementation was found to restore the reduced levels of α-KG, increase TET demethylase activity and normalize methylation patterns that were observed to be hypermethylated in cardiac mesenchymal stem cell (CMSCs) from diabetic individuals (Spallotta et al., 2018). Importantly, the functional and clinical outcomes of α-KG-induced epigenetic modifications on stem cells have been shown to differ between species and cell types, indicating that the consequences of α-KG-induced epigenetic modifications are dependent on its context. α-KG supplementation induced maintenance of pluripotency in mouse naive embryonic stem cells (ESC) by upregulating TET and KDM enzymatic activities (Carey et al., 2015), whereas α-KG supplementation was shown to promote differentiation in primed human ESC (TeSlaa et al., 2016).

Both fumarate and succinate compete with α-KG for the binding pocket of the Fe(II)/α-KG dependent dioxygenases, including TET and KDM demethylases, thereby altering the epigenetic landscape of several mammalian cells (Figure 4). Succinate-induced inhibition of KDMs and TETs was shown to initiate histone and DNA hypermethylation, which had large effects on the expression of genes that regulate cancer cell progression (Cervera et al., 2009; Killian et al., 2013; Letouze et al., 2013; Mason and Hornick, 2013) and the induction of epithelial-to-mesenchymal transition (EMT) (Aspuria et al., 2014). In a similar fashion, accumulating fumarate induced hypermethylation of a class of anti-metastatic miRNAs (miR-200) in fumarate hydratase (FH)-deficient renal cancer cells (Sciacovelli et al., 2016). Whereas miR-200 normally suppressed transcription factors that mediate EMT initiation (Craene and Berx, 2013), fumarate-induced hypermethylation of miR-200 prevented this suppression and activated the EMT (Sciacovelli et al., 2016). Combined, mitochondrial-derived α-KG, fumarate and succinate have been shown in cancer cells and stem cells to drive the nuclear epigenetic landscape.

#### B-Vitamin Regulation of Histone and DNA Methylation

Vitamin B2, in the form of FAD, is a co-factor for the lysine demethylase, KDM1, which is an α-KG-independent histone demethylase with amine oxidase activity (Maes et al., 2015). KDM1 lysine demethylases are involved in demethylation of H3K4 and H3K9, which are associated with transcriptional repression and activation, respectively (Shi et al., 2004; Maes et al., 2015). One of these KDM1 family members, the lysine demethylase (KDM1A or lysine-specific histone demethylase 1A (LSD1)), is particularly sensitive to FAD availability (Figure 4) (Hino et al., 2012; Liu and Zempleni, 2014b,a). FAD availability was found to alter histone methylation in adipocytes (Hino et al., 2012). Silencing of riboflavin kinase and FADS inhibited KDM1A demethylase activity, resulting in increased methylation of histones and loss of repression of genes related to energy expenditure and ultimately to increased mitochondrial respiration and the induction of lipolysis (Hino et al., 2012). Impaired KDM1A demethylase activity due to vitamin B2 deficiency was also found to skew immune cells toward a pro-inflammatory phenotype (Liu and Zempleni, 2014a). Vitamin B2 deficiency resulted in an increased methylation of histones on genes encoding pro-inflammatory cytokines, like tumor necrosis factor-alpha and IL-1beta, highlighting a role for vitamin B2 deficiency in immune signaling (Liu and Zempleni, 2014a).

Methylation of histones and DNA requires methyl-donors. The B-vitamins, B2, B6, B11 (folate) and B12 (cobalamin), are necessary to produce the methyl donor SAM from pathways that drive one-carbon metabolism (Figure 4). The folate and methionine cycle consist of a complex set of reactions operating in both the mitochondria and the cytosol. Mitochondria-derived serine is the major precursor for the methionine that forms SAM (Ducker and Rabinowitz, 2017). SAM is a substrate for nuclear histone and DNA methylase enzymes that transfer the methyl group of SAM to histone lysines or DNA cytosines. Vitamin B11 (folate) deficiency leads to neural tube defects, which can be attributed to impaired DNA synthesis, but has also been shown to be associated with alterations in the methylation landscape during embryonic development of the brain (Chang et al., 2011). In adult humans, lowered folate intake was associated with DNA hypomethylation in lymphocytes (Jacob et al., 1998).

In a study using human embryonic stem cells (hESCs), it was shown that nicotinamide-N-methyl transferase (NNMT) expression keeps hESC in a pluripotent state, by consuming methyl donors, lowering SAM levels and concomitantly lowering methylation of H3K27 at specific loci (Sperber et al., 2015). Among the loci affected were the EGLN1 gene as well as genes from the Wnt signaling pathway. Interestingly, the observed mechanism integrates multiple aspects of B-vitamin regulation of mito-nuclear signaling. NNMT not only lowers the availability of the methyl donor SAM, it also lowers the availability of nicotinamide for NAD^+^ synthesis. Since NAD^+^ availability also impacts histone acylation via SIRT regulation, this could imply that a crosstalk exists between regulation of the synthesis of NAD^+^ and SAM, to maintain methylation and acylation epigenetic states in the nucleus. Furthermore, the NNMT mechanism of maintaining pluripotency also demonstrates an interaction between histone methylation status and HIF1 signaling via regulation of the EGLN locus (Sperber et al., 2015). This interaction between nuclear methylation and HIF1 signaling was also demonstrated to occur via FAD (vitamin B2) regulation of KDM1A, where FAD regulated HIF1A stability in a KDM1-dependent fashion in cancer cells (Yang et al., 2017).

Vitamin B12 (cobalamin) is involved in the methionine cycle through its role as an essential co-factor for the MS protein (Figure 4). Maternal cobalamin status has been linked to methylation status at specific loci in the offspring, also a weak association between maternal cobalamin status and child’s cognition was observed (Caramaschi et al., 2017). Furthermore, in a mouse model of reduced cobalamin import into the brain generated by knocking out the CD320 cobalamin receptor, global brain DNA hypomethylation was observed (Fernandez-Roig et al., 2012; Lai et al., 2013). Vitamin B6 is mainly shown to regulate DNA synthesis via its role in one-carbon metabolism (Depeint et al., 2006a), but dietary vitamin B6 intake was also shown to be linked to hypermethylation of the MLH1 promotor in colorectal tumors in humans (de Vogel et al., 2008).

Similar to the role of the B-vitamins in regulating EGLNs via TCA cycle intermediates in HIF1 signaling, altering TCA cycle intermediates could also impact DNA and histone methylation status via the regulation of the TET and KDM demethylases. Evidence for a direct role of B-vitamins on nuclear methylation via TCA cycle intermediates is lacking. Likely, because both vitamin B1 and vitamin B2 are directly involved in regulating either methyl donor availability or FAD-dependent histone demethylase activity, respectively. However, maintaining TCA cycle function by vitamin B1, B2, and B3 is likely to also play a role in regulating DNA and histone methylation in the nucleus (Figure 4).

### Regulation of Histone Acylation

**FIGURE 5:**
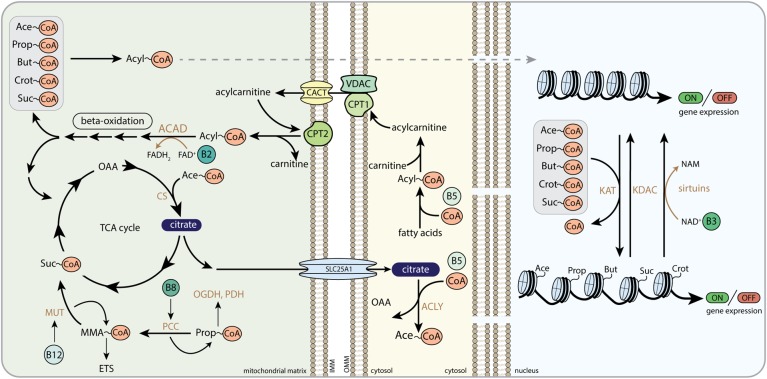
The role of B-vitamins in acyl-CoA metabolism and histone acylation. Acyl-CoA transport from the mitochondria to the nucleus, here depicted as dashed arrow, follows multiple different routes as described in the text. CoA, Coenzyme A; Ace-CoA, acetyl-CoA; Prop-CoA, propionyl-CoA; But-CoA, butyryl-CoA; Crot-CoA, crotonyl-CoA; Suc-CoA, succinyl-CoA; FAD, flavin adenine dinucleotide; FADH_2_, hydroquinone form of FAD; NAD^+^, nicotinamide adenine dinucleotide; NADH, reduced form of NAD^+^; NAM, nicotinamide; OAA, oxaloacetate; CS, citrate synthase; MUT, methylmalonyl-CoA mutase; MMA-CoA, methylmalonyl-CoA; ETS, electron transfer system; PCC, propionyl-CoA carboxylase; OGDH, 2-oxoglutarate dehydrogenase complex; PDH, pyruvate dehydrogenase complex; SLC25A1, mitochondrial tricarboxylate transport protein; ACLY, ATP-citrate lyase; ACAD, acyl-CoA dehydrogenase; CPT1, carnitine palmitoyltransferase 1; CPT2, carnitine palmitoyltransferase 2; VDAC, voltage-dependent anion channels; CACT, carnitine/acylcarnitine translocase; KAT, lysine acetyltransferase; KDAC, lysine deacetylase; OMM, outer mitochondrial membrane; IMM, inner mitochondrial membrane.

Citrate was one of the first mitochondrial metabolites that was shown to serve metabolic, as well as non-metabolic functions outside mitochondria. In the 1950s, it was demonstrated that citrate is not only oxidized for ATP generation in mitochondria, but also stimulates fatty acid synthesis in the cytosol (Brady and Gurin, 1951; Srere, 1965). Citrate is generated from acetyl-CoA and OAA condensation catalyzed by the mitochondrial enzyme citrate synthase (CS). Mitochondrial-derived citrate can be exported to the cytosol by the citrate carrier (SLC25A1) where citrate can be regenerated to acetyl-CoA and OAA by ATP citrate lyase (ACLY) (Figure 5). Whereas OAA is shuttled back into the mitochondria in the form of malate, citrate-derived acetyl-CoA can subsequently be used to fuel anabolic reactions, such as biosynthesis of fatty acids, amino acids and steroids (Iacobazzi and Infantino, 2014; Pietrocola et al., 2015).

In addition to serve as an anabolic intermediate and cytosolic signaling molecule, citrate provides a source of nuclear acetyl-CoA for histone acetyltransferase (HAT or lysine acetyltransferase; KAT) activity and promotes acetylation reactions in the nucleus (Figure 5) (Wellen et al., 2009). In histone acetylation, the cleaved acetyl-group from acetyl-CoA is transferred to an ε-*N*-lysine residue of histones in chromatin structures to produce ε-*N*-acetyllysine residues (Montgomery et al., 2015). Histone lysine acetylation plays a pivotal role in nuclear gene expression (Verdin and Ott, 2015). Addition of negatively charged acetyl-groups neutralizes the positively charged lysine residues on the histone tails. This unlocks the tight interactions between negatively charged DNA and positively charged histones and allows transcription factor binding. Multiple studies have demonstrated that nuclear and cytosolic acetylation is dependent on citrate efflux from the mitochondria, since loss of enzymes that generate, transport or cleave citrate hampers cytosolic acetyl-CoA production and protein acetylation (Morciano et al., 2009; Wellen et al., 2009; Yi et al., 2011; Londono Gentile et al., 2013; Ashbrook et al., 2015; Sivanand et al., 2017; Lee et al., 2018). For example, ACLY-dependent acetylation was recently found to induce the expression of cell migration and adhesion genes, promoting malignant tumor formation (Lee et al., 2018). Silencing ACLY was also shown to decrease histone acetylation in several mammalian cell types (Wellen et al., 2009; Sivanand et al., 2017), indicating that mitochondrial-derived citrate plays a role in tumorigenesis by indirect alterations in transcriptional programs that dictate cancer cell formation and progression. Acetate derived acetyl-CoA in the nucleocytoplasmic compartment was found to rescue histone acetylation in ACLY deficient colon cancer cells, also indicating that acetyl-CoA can also be produced from extra-mitochondrial citrate and acetate (Wellen et al., 2009).

Fatty acids are a major source of acetyl-CoA as well as other acyl-CoAs, which form the acyl donors for histone acylation (Figure 5). Acyl-CoAs can be generated in different compartments of the cell, and mitochondrial-derived acyl-CoAs have been shown to be a source for histone acylation, allowing for metabolic control of histone acylation and gene transcription by acyl-CoA molecules from inside the mitochondria. Export of acyl-CoAs out of the mitochondria is mediated by reversing parts of the machinery of the carnitine shuttle. Carnitine palmitoyltransferase 2 (CPT2) is able to convert medium and long-chain acyl-CoAs into acylcarnitines (Violante et al., 2013), whereas an additional enzyme called carnitine acetyl transferase (CRAT) takes care of the conversion of short-chain acyl-CoAs into acylcarnitines (Violante et al., 2013). Acylcarnitines are likely to be translocated over the mitochondrial inner membrane by carnitine-acylcarnitine translocase (CACT), making them available for the cytosol and the nucleus.

Although histone acetylation is the major contributor to chromatin regulation and is partly regulated by mitochondrial acetyl-CoA levels (Moussaieff et al., 2015), recent compelling data have shown that histones are modified by a variety of acylation reactions (Figure 5) (Tan et al., 2011). Among them, propionylation, butyrylation, crotonylation, hydroxy-isobutyrylation and succinylation have now been characterized biologically to some extent as well. Propionylation at H3K14 was enriched at transcription start sites and promotors in mouse livers (Kebede et al., 2017). H3K14 propionylation marks overlapped substantially with active H3K9Ac and H3K4me3 marks, providing opportunities for more sophisticated recruitment of transcriptional regulators (Kebede et al., 2017). Interestingly, histone propionylation levels were altered by deleting the mitochondrial propiony-CoA generating enzyme propionyl-CoA carboxylase, demonstrating that mitochondrial derived propionyl-CoA impacts nuclear histone propionylation (Kebede et al., 2017). In another study, increased H3K14 propionylation induced by elevated propionyl-CoA in nuclear extracts lowered H3K14 acetylation levels (Simithy et al., 2017), highlighting the dynamic interplay between different lysine acylation modifications via acyl-CoA substrate levels. Other histone sites have also been shown to be functionally propionylated. Overexpression of a newly identified propionyltransferase (MOF) increased histone propionylation levels on multiple histone marks, such as H4K16, H4K12, H2Ak5, and H2K9 (Han et al., 2018), and H3K23 propionylation levels were lowered upon monocytic differentiation in cultured U937 cells (Liu et al., 2009). Butyrylation of histones was shown to play a role in dynamically regulating genome organization during spermatogenesis, by mediating Brdt bromodomain binding to histone marks (Goudarzi et al., 2016). Brdt recognized acetylated H4K5 sites but did not bind to butyrylated sites on the same H3K5 lysines (Goudarzi et al., 2016), again showing the interplay between different acylations.

Histone crotonylation is different from other short-chain acylation modification in that the crotonyl modification contains an unsaturated bond, making this modification more rigid and thus could exert unique biological functions. Crotonyl-CoA is a mitochondrial fatty acid oxidation intermediate generated in the first step of butyryl-CoA oxidation, which is in turn derived mainly from longer chain fatty acyl-CoAs. Short-chain acyl CoA dehydrogenase (SCAD) catalyzes the reaction making the *trans*-2-enoyl-CoA from the short-chain fatty acyl-CoA. Moreover, crotonyl-CoA is formed from glutaryl-CoA during tryptophan and lysine degradation by glutaryl-CoA dehydrogenase (GCDH). Although crotonyl-CoA is generally converted by the enoyl-CoA hydratase, crotonase, into 3-hydroxybutyryl-CoA it can also be converted to crotonylcarnitine and/or crotonate. It is likely that mitochondrial crotonyl-CoA can be a source for nuclear histone lysine crotonylation, but it has not been directly established yet. Histone crotonylation was first discovered by the group of Yingming Zhao (Tan et al., 2011), using sensitive mass spectrometry proteomics techniques and was demonstrated to mark X-linked genes that are post-meiotically expressed in round spermatids (Tan et al., 2011). In stages of late meiosis during spermatogenesis most of the genes are silenced, but a selection of genes can become activated after meiosis. Mostly the genes associated with the histone crotonylation marks are enriched for escaping chromosome inactivation (Tan et al., 2011). Again, it has been shown in multiple studies that dynamically regulating the levels of crotonyl-CoA, also regulates the levels of histone crotonylation (Sabari et al., 2015). Incubating cultured cells with high concentrations of short-chain fatty acids, generally increases respective lysine modifications globally in the cell (Pougovkina et al., 2014; Colak et al., 2015). In line, incubating RAW264.7 macrophages with crotonate, increased histone crotonylation, and at the same time activated the excretion of cytokines upon LPS stimulation, which differentiates macrophages into a pro-inflammatory state (Sabari et al., 2015). This activation could be turned off when the enzyme that is likely responsible for turning crotonate into crotonyl-CoA (acetyl-CoA synthetase 2, ACSS2) was knocked down (Sabari et al., 2015), demonstrating that crotonyl-CoA levels are drivers of the activated gene expression signature in RAW264.7 macrophages. Similar studies have been performed for the novel histone modification lysine beta-hydroxybutyrylation, where metabolic states of increased beta-hydroxybutyrate where associated with specific increases in histone beta-hydroxybutyrylation marks (Xie et al., 2016). In an effort to identify novel histone crotonyltransferase enzymes, Liu et al. (2017) discovered that the chromodomain protein CDYL lowers histone crotonylation instead of increases it, hinting at a different function for this protein than a histone crotonyltransferase. Intriguingly, the CDYL protein possessed enoyl-CoA hydratase activity, which allowed CDYL to convert crotonyl-CoA into hydroxybutryl-CoA and thereby lowering the available pool of crotonyl-CoA for histone crotonylation (Liu et al., 2017). Thus, apart from mitochondrial regulation of acyl-CoA levels, a fine-tuning machinery likely exist inside the nucleus to regulate crotonyl-CoA levels as well as levels of other acyl-CoAs.

Lysine succinylation was first discovered to be primarily a modification that occurs inside the mitochondria (Du et al., 2011; Park et al., 2013; Rardin et al., 2013), other studies soon identified lysine succinylation in the cytosol and the nucleus as well (Kumar and Lombard, 2018). The first succinylation on histone peptides in yeast and mammalian cells were discovered by Xie et al. (2012) and functional consequences of altering histone lysines with dicarboxylic acid modification, like succinylation, were identified more recently (Li L. et al., 2016; Smestad et al., 2018). H3K122 succinylation was shown to play a role in regulating the DNA-damage response (Li L. et al., 2016). SIRT7, a NAD-dependent deacylase protein from the SIRT family, was identified as histone desuccinylation enzyme (Li L. et al., 2016). SIRT7 was recruited to DNA double-strand breaks, where SIRT7 desuccinylated H3K122, which ultimately promoted DNA damage repair and cell survival (Li L. et al., 2016). SIRT7 has already been shown extensively to have multiple roles in nuclear processes, but it was previously only understood to exert its action by lysine deacetylation, instead of lysine desuccinylation (Chalkiadaki and Guarente, 2015).

That mitochondrial alteration of succinate and succinyl-CoA levels could impact nuclear histone succinylation directly, was shown by Smestad et al. (2018). Mouse embryonic fibroblasts deficient for SDH accumulated succinate and succinyl-CoA (Smestad et al., 2018). This in turn elevated global lysine succinylation levels and altered histone lysine succinylation distributions (Smestad et al., 2018). Histone succinylation was specifically enriched 600 bp from TSS and was abundant at highly expressed genes. Moreover, in line with the observed DNA damage response defect in the SIRT7 deficient cells (Li L. et al., 2016), SDH deficient cells presented with elevated DNA damage, as was shown by analyzing phospho-γH2A.X, making them more sensitive to the genotoxic drugs, etoposide, and gemcitabine (Smestad et al., 2018). Although it is currently not precisely known how succinate from the mitochondria is converted into succinyl-CoA in the nucleus, because succinyl-CoA synthetase activities have not been clearly established in nucleo/cytosolic compartments, other succinyl-CoA generating enzymes are mainly localized in mitochondria and peroxisomes, and succinylcarnitine is not a substrate for nucleo/cytosolic acetyltransferases, this study does highlight the important role of mitochondrial signaling to the nucleus via mitochondrial generated metabolites. An alternative nuclear succinyl-CoA source was shown to be α-KG (Wang et al., 2017). The OGDH complex, which was previously thought to only reside in the mitochondria, was demonstrated to be present in the nucleus of mammalian cells and was bound and recruited by the histone acetyltransferase GCN5 (KAT2A) to histones (Wang et al., 2017). This mechanism allows for the local production of succinyl-CoA from the mitochondrial-derived metabolite α-KG.

#### B-Vitamin Regulation of Histone Acylation

Already in 1948 it was demonstrated that vitamin B5 (pantothenate) deficiency in rats causes decreased levels of acetylation of p-aminobenzoic acid (PABA) (Riggs and Hegsted, 1948), but experimental evidence that vitamin B5 could regulate histone acylation is scarce. In a study using drosophila, histone acetylation has been linked with decreased availability of CoA (Siudeja et al., 2011). By interfering with pantothenate kinase activity, either via chemical inhibition or genetic knockdown in drosophila and mammalian cell models, these authors showed that *de novo* CoA biosynthesis is essential to support histone and tubulin acetylation (Siudeja et al., 2011). Since other studies have demonstrated that reduced levels of histone and tubulin acetylation are associated with neurodegeneration, it was speculated that at least part of the neurodegeneration observed in pantothenate kinase-associated neurodegeneration (PKAN) might be attributed to altered protein acetylation states that occur as a consequence of impaired CoA metabolism (Siudeja et al., 2011). In a follow-up study, addition of extracellular CoA was able to reverse the effects of decreased CoA availability on histone acetylation (Srinivasan et al., 2015), demonstrating that increasing CoA levels could impact histone acetylation.

Other critical regulators of lysine acylation that are B-vitamin sensitive are the vitamin B3 (NAD^+^)-dependent sirtuins (Figure 5), with SIRT3-5 primarily localized in the mitochondria and SIRT1, SIRT2, SIRT6, and SIRT7 primarily localized in the nucleocytoplasmic compartment, although other sirtuins have been proposed to reside in the nucleus as well (Iwahara et al., 2012). SIRT1, SIRT6, and SIRT7 have a plethora of nuclear proteins targets, especially those affecting metabolism directly or indirectly (Haigis and Sinclair, 2010; Chalkiadaki and Guarente, 2012). SIRT1 deacetylates multiple histones directly in a NAD-dependent fashion (Zhang and Kraus, 2010). SIRT1 regulation of histone acetylation could program the epigenetic code and play an important role in chromatin regulation. SIRT1 redistributes to double strand breaks upon DNA damage, promote repair and alter gene expression (Oberdoerffer et al., 2008). Interestingly, among its many nuclear targets, SIRT1 also regulates the EP300-acetyltransferase which functions as a general histone acetyltransferase (Zhang and Kraus, 2010). Also, SIRT6 and SIRT7 are able to deacetylate histone targets making them important regulators of many cellular activities, such as cell proliferation, ribosome biogenesis, metabolic homeostasis, and DNA damage repair (Blank and Grummt, 2017; Tasselli et al., 2017).

Since fatty acid oxidation provides many of the acyl-CoAs needed for histone acylation, B-vitamins involved in regulation of fatty acid oxidation could play a role in regulating acylation in general and histone acylation in specific. Mitochondrial acyl-CoA dehydrogenases, the enzymes that perform the first step in fatty acid beta-oxidation, are flavoproteins that require FAD and thus vitamin B2 (riboflavin) (Figure 5). Levels of multiple acyl-CoA dehydrogenases, mitochondrial CoA pools, as well as the levels of fatty acid derivatives are altered in riboflavin deficiency (Olpin and Bates, 1982; Veitch et al., 1988; Nagao and Tanaka, 1992). The sensitivity of these metabolic enzymes for FAD deprivation cannot only have consequences for mitochondrial lipid metabolism, but are also likely to alter mito-nuclear signaling pathways that are modulated by acyl-CoAs. Using a proteomic approach on livers of Pekin Ducks, Tang et al. (2017) identified that especially mitochondrial enzymes involved in lipid metabolism and respiration are sensitive to vitamin B2 deficiency (Tang et al., 2017), whereas the impact of vitamin B2 deficiency on histone acylation was not studied.

Biotin (vitamin B8/B7) facilitates the metabolic reaction that is catalyzed by propionyl-CoA carboxylase, which converts propionyl-CoA into methylmalonyl-CoA in the mitochondria (Figure 5). Deficiency of biotin leads to elevated levels of mitochondrial CoA intermediates, including 3-methylcrotonyl-CoA and propionyl-CoA (Liu et al., 1993; Hernandez-Vazquez et al., 2013). Accumulating levels of propionyl-CoA can induce mitochondrial toxicity by inhibiting PDH and OGDH and impairing the activity of CIII within the ETS (Schwab et al., 2006) (Figure 5). Although the impact of biotin deficiency on nuclear acylation reactions, such as propionylation, have not been studied so far, alterations in biotin availability are likely to alter cellular protein propionylation levels and could impact the dynamic interplay between multiple acylation reaction on histones, such as histone acetylation and propionylation.

The levels of methylmalonyl-CoA are elevated upon a deficiency in cobalamin (vitamin B12), which is the co-factor for MUT (Figure 5). Cobalamin deficiency is therefore characterized by accumulating levels of MMA in plasma and urine (Chandler et al., 2009). Several studies reported that MMA impairs mitochondrial function by acting as a mitochondrial toxin that inhibits SDH (Toyoshima et al., 1995; Narasimhan et al., 1996). Whereas some studies did not find MMA-induced SDH inhibition when MUT was dysfunctional (Krahenbuhl et al., 1990), other studies suggested that instead of MMA, alternative metabolites that accumulate upon MUT deficiency (2-methylcitric acid, malonic acid, and propionyl-CoA) synergistically induce mitochondrial dysfunction (Kolker et al., 2003). Other complexes of the ETS were also reported to be inhibited in MUT deficiency (Krahenbuhl et al., 1991; Brusque et al., 2002; Chandler et al., 2009). Interestingly, other new acyl-CoAs were recently also linked to cobalamin and MUT (Shen et al., 2017). Itaconyl-CoA and citramalyl-CoA both accumulated in cells with a mutation in citrate lyase subunit beta (CLYBL), leading to impaired mitochondrial cobalamin metabolism (Shen et al., 2017). Mechanistically, itaconyl-CoA and citramalyl-CoA impair MUT activity by poisoning adenosylcobalamin, which cannot be regenerated and induces cobalamin deficiency (Shen et al., 2017). Again, these acyl-CoAs could also interfere with other more abundant histone acylation marks or could act as novel PTMs on histones, giving that they are able to reach the nucleus. Thus, cobalamin deficiency could affect mito-nuclear communication via regulation of specific acyl-CoA species.

### ROS and Redox Signaling

**FIGURE 6:**
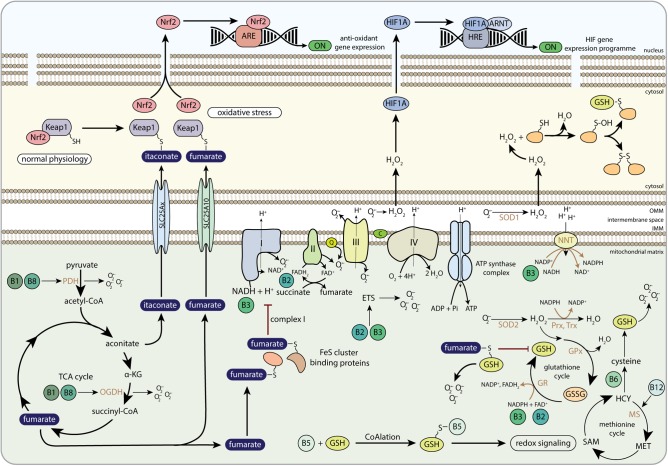
The role of B-vitamins in redox metabolism and redox-related mito-nuclear communication. Keap1, Kelch-like ECH-associated protein 1; Nrf2, nuclear factor-like 2; ARE, antioxidant response element; H_2_O_2_, hydrogen peroxide; HIF1A, hypoxia-inducible factor 1 alpha; ARNT, aryl hydrocarbon receptor nuclear translocator; HRE, hypoxia response element; PDH, pyruvate dehydrogenase; α-KG, alpha-ketoglutarate; OGDH, 2-oxoglutarate dehydrogenase complex; SLC25Ax, SLC25A1, SLC25A10, or SLC25A11; SLC25A10, mitochondrial dicarboxylate carrier; FeS cluster, iron-sulfur cluster; FAD, flavin adenine dinucleotide; FADH_2_, hydroquinone form of FAD; NAD^+^, nicotinamide adenine dinucleotide; NADH, reduced form of NAD^+^; Q, coenzyme Q; C, cytochrome C; NNT, nicotinamide nucleotide transhydrogenase; NADP^+^, nicotinamide adenine dinucleotide phosphate; SOD1, copper-zinc superoxide dismutase; SOD2, manganese-dependent superoxide dismutase; Prx, peroxiredoxins; Trx, thioredoxins; GPx, glutathione peroxidase; GR, glutathione reductase; GSSG, glutathione disulphide; GSH, glutathione; ETS, electron transfer system; HCY, homocysteine; MS, methionine synthase; MET, methionine; SAM, *S*-adenosylmethionine; OMM, outer mitochondrial membrane; IMM, inner mitochondrial membrane.

Reactive oxygen species (ROS) are highly reactive biomolecules that are able to react with lipids, DNA and proteins. They are produced as a result of mitochondrial metabolism but also by other enzymes such as NADPH oxidases (Figure 6). Cells also possess powerful anti-oxidant systems to detoxify ROS, and the balance between ROS production and detoxification determines the cellular redox state. It is nowadays known that this cellular redox state plays an important role in cellular (patho)physiology (Holmstrom and Finkel, 2014). Regulating vascular tonality, enzyme activity, gene expression, cellular proliferation and differentiation are a few physiological examples that can be modulated by ROS (McDonald and Murad, 1995). Increased ROS production and/or decreased detoxification is linked to aging (Finkel and Holbrook, 2000) as well as pathological conditions such as cancer, Alzheimer’s disease and diabetes (Freeman et al., 2006; Kumar et al., 2008; Pi and Collins, 2010; Sabens Liedhegner et al., 2012).

One of the main functions of mitochondria is the production of ATP to drive cellular processes. Mitochondrial ATP generation is ensured by the ETS consisting of 4 multi-protein complexes, which together with the F1Fo-ATP synthase (CV) forms the oxidative phosphorylation (OXPHOS) system (Figure 6). The function of the ETS is to generate an electrochemical gradient over the inner mitochondrial membrane by pumping protons from the matrix to the inter membrane space, which is needed to generate ATP via CV (Mitchell, 1961). To perform this function, the ETS takes up electrons from NADH at CI or succinate, with FADH_2_ as an intermediate, at CII, which are transferred to CIII via ubiquinone (Figure 6). Cytochrome c transfers the electrons from CIII to CIV where they react with oxygen to form water. Most literature states that 0.5–2% of the electrons leak out of the ETS and react with oxygen to form ROS (Boveris, 1977; Goncalves et al., 2015). Superoxide (O_2_^-^) and hydrogen peroxide (H_2_O_2_) can be formed at the FMN site in CI (Kussmaul and Hirst, 2006; Grivennikova and Vinogradov, 2013) and CII (Quinlan et al., 2012; Siebels and Drose, 2013; Jardim-Messeder et al., 2015) and superoxide is also formed at the quinol-oxidizing site of CI and CIII and the FeS cluster of CIII (Figure 6) (Muller et al., 2003; Lambert and Brand, 2004; Grivennikova and Vinogradov, 2013). Although ROS production by the ETS is clearly demonstrated, the ETS is not the only source of ROS in mitochondria. Two other mitochondrial proteins playing an important role in ROS formation are PDH and OGDH, as they both contain a similar flavosubunit that can be a source of superoxide (Figure 6) (Starkov et al., 2004; Fisher-Wellman et al., 2013; Quinlan et al., 2014). Other mitochondrial proteins involved in ROS production are aconitase (Gardner, 2002; Ternette et al., 2013), *S*(*n*)-glycerol-3-phosphate dehydrogenase (Orr et al., 2012), dihydroorotate dehydrogenase (Forman and Kennedy, 1975; Orr et al., 2012), monoamine oxidases and p66shc/cytochrome-c (Giorgio et al., 2005; Di Lisa et al., 2009). However, how much a protein contributes to the total production of mitochondrial ROS depends on the substrates being used by the mitochondria. Still, in most cases CI, CII, and CIII combined has the highest contribution, varying between 75 and 100% of the total mitochondrial ROS production (Quinlan et al., 2013).

To act as a metabolic signal in the nucleus, mitochondrial ROS should travel a certain distance and pass various membranes. Superoxide is negatively charged and is therefore unlikely to pass the (mitochondrial) membranes, although it might use the voltage dependent anion channel to diffuse to the cytoplasm (Han et al., 2003). Furthermore, the diffusion capacity of superoxide is affected by superoxide dismutase (SOD) activity, which can reduce the lifetime of superoxide from 100 ms to 35 μs (Mikkelsen and Wardman, 2003; Cardoso et al., 2012), thereby limiting the traveling distance to 400 nm. Hydrogen peroxide is uncharged and shows physical characteristics similar to water and can therefore freely diffuse through membranes, depending on its form and composition and the presence of aquaporins (Mathai and Sitaramam, 1994; Antunes and Cadenas, 2000; Sousa-Lopes et al., 2004; Bienert et al., 2007). More importantly, many anti-oxidant systems limit the diffusion distance. When taking the cellular concentrations of peroxiredoxin (Prx) 2, glutathione peroxidase (Gpx) 1 and GSH into account, the diffusion distance is estimated to be 4, 6, and 1600 μm, respectively (Winterbourn and Hampton, 2008). Taken together, mitochondrial ROS might not always be able to directly exert nuclear signaling or signaling is limited to perinuclear located mitochondria. Interestingly, it has been proposed that in plant cells, hydrogen peroxide can diffuse over distances up to 10 μm at a frequency up to 2 Hz (Vestergaard et al., 2012), implying that hydrogen peroxide potentially encodes a cellular message depending on amplitude, frequency and localization. The observation that oscillations of the mitochondrial membrane potential are dependent on cellular redox status, suggests that mitochondria could act as relay stations to send out a ROS-mediated message throughout the cell (Schwarzlander et al., 2012). How this eventually is transformed into a cellular response remains unknown.

But how can mitochondria and mitochondrial metabolites affect nuclear signaling via redox balance? One well-described way to affect nuclear signaling is via the stabilization of HIF1A. *In vivo*, the mitochondrial ETS was shown to be essential for HIF1A stabilization and further *in vitro* studies indicated that CIII-derived mitochondrial ROS played an important role in stabilizing HIF1A (Hamanaka et al., 2016). Importantly, these effects can be reversed by overexpressing catalase (CAT) and GPx, but not mitochondrial targeted-CAT, SOD1 and SOD2 (Chandel et al., 2000; Brunelle et al., 2005; Guzy et al., 2005), suggesting that superoxide formed at CIII needs to be converted into hydrogen peroxide in the cytoplasm before it can stabilize HIF1A levels (Figure 6). Although the exact mechanism by which HIF1A is stabilized is still not clear, EGLNs are most likely involved, because ROS competes with oxygen in EGLNs, which stalls the hydroxylation reaction and results in stabilization of HIF1A.

Fumarate is able to regulate ROS levels by interfering with anti-oxidant systems such as GSH (Sullivan et al., 2013). Sullivan et al. (2013) were the first to show that fumarate, which accumulated due to fumarate hydratase deficiency, can induce succination of GSH, generating succinated GSH (succinic-GSH) (Figure 6). Succinic-GSH would support cancer cell proliferation by increasing oxidative stress levels in the mitochondria that subsequently activate HIF1A (Sullivan et al., 2013). Mechanistically, succinic-GSH acts as an alternative substrate for GR, thereby lowering NADPH levels, which limits the pool of NADPH that is available to be used as a cofactor for hydrogen peroxide detoxification (Sullivan et al., 2013). Two years later, this mechanism was argued by Zheng et al. (2015). Although they agreed that succinic-GSH-induced oxidative stress was the leading cause for the clinical manifestations of FH-deficiency, they argued against the mechanism by which succinic-GSH and concurrent NADPH depletion enhanced oxidative stress (Zheng et al., 2015). Instead of detoxification of succinic-GSH by GR as an enhancer of oxidative stress, Zheng et al. (2015) demonstrated that succinic-GSH depleted the cells of GSH, and that NADPH requirements were increased to sustain GSH biosynthesis. These redox imbalances were not only relevant for anti-oxidative defense, but also for cancer cell fate (Zheng et al., 2015).

Apart from GSH succination, fumarate can also have a signaling role via its ability to act as substrate for succination of other proteins (Adam et al., 2011; Merkley et al., 2014; Yang et al., 2014). Multiple proteins are subjected to succination (Figure 6) (Bardella et al., 2011; Merkley et al., 2014; Yang et al., 2014), including Kelch-like ECH-associated protein 1 (KEAP1) (Kaspar et al., 2009), aconitase (Ternette et al., 2013), iron regulatory protein 1 (IRP1) (Kerins et al., 2017), FeS cluster binding proteins (Tyrakis et al., 2017), and glyceraldehyde-3-phosphate dehydrogenase (GAPDH) (Blatnik et al., 2008; Kornberg et al., 2018). Succination leads to protein dysfunction and disruption of redox homeostasis (Sullivan et al., 2013; Zheng et al., 2015) and therefore contributes to the pathology of various chronic diseases, including cancer (Adam et al., 2011; Sullivan et al., 2013; Zheng et al., 2015; Menegon et al., 2016; Kerins et al., 2017) and diabetes (Alderson et al., 2006; Frizzell et al., 2009; Adam et al., 2017). In cancer, elevated levels of fumarate increased succination of KEAP1, impairing its function, which induced nuclear translocation of nuclear factor-like 2 (Nrf2) and thereby activating antioxidant response element (ARE)-controlled genes to neutralize oxidative stress and to create an advantageous growth environment for cancer cells (Figure 6) (Ooi et al., 2011; Menegon et al., 2016). Remarkably, although fumarate-induced Nrf2 stabilization lead to adverse effects in the kidney, the upregulation of ARE-controlled genes exhibits cardioprotective properties in the heart (Ashrafian et al., 2012). Furthermore, via concurrent activation of Nrf2 and succination of IRP1, fumarate was found to enhance transcription and translation of the ferritin gene, respectively. As a consequence, ferritin levels increased, which promoted the expression of promitotic transcription factor Forkhead box protein M1 (FOXM1), inducing cell cycle progression (Kerins et al., 2017).

Another PTM in the KEAP1-Nrf2 pathway, is also derived from mitochondrial metabolism. Mills et al. (2018) demonstrated that itaconate alkylated cysteine residues of KEAP1 in LPS-activated macrophages (Mills et al., 2018). Whereas KEAP1 prevents nuclear migration of Nrf2 by forming a cluster under normal physiological conditions, alkylation of KEAP1 by itaconate separates Nrf2 from KEAP1 (Figure 6) (Mills et al., 2018), liberating Nrf2 for migration to the nucleus, where it activates the transcription of genes involved in anti-oxidant and anti-inflammatory signaling routes (Hayes and Dinkova-Kostova, 2014; Mills et al., 2018). Bambouskova et al. (2018) also identified an anti-inflammatory role of itaconate in cells. Itaconate inhibited the production of a subset of pro-inflammatory cytokines IL-6 and IL-12 by inhibiting nuclear IkBζ, a key player in the secondary transcriptional response upon primary NF-κB activation (Bambouskova et al., 2018). Although the anti-inflammatory role of itaconate via its nuclear signaling routes have only recently been discovered, these studies highlight the important role of the aconitate-derived itaconate in mito-nuclear communication in immune cells.

#### B-Vitamin Regulation of ROS and Redox Signaling

Vitamin B2 and B3 act as cofactors for, respectively, CII and CI, and assist in redox reactions catalyzed by GR, in which GSSG is reduced to GSH. Vitamin B2 and B3 are therefore regulators of ROS balance, and dysregulated vitamin B2 or B3 metabolism leads to increased levels of oxidative stress and mitochondrial ROS production (Figure 6) (Ashoori and Saedisomeolia, 2014; Arias-Mayenco et al., 2018). Amongst the signaling pathways that are affected by mitochondrial ROS, especially lipid peroxidation and oxidative stress injuries that are caused by I/R, have been found to increase upon vitamin B2 deficiency (Ashoori and Saedisomeolia, 2014; Sanches et al., 2014). Furthermore, chronic supplementation with the vitamin B3 (in the form of NAM) was shown to reduce hepatic lipid accumulation *in vivo*, which was suggested to ameliorate the oxidative stress response that occurred during liver steatosis (Mitchell et al., 2018). Similarly, NR has been shown to lower oxidative stress in mice in an LPS-induced sepsis model, resulting in lower mortality (Hong et al., 2018). Although multiple studies have shown beneficial effects of NR (Yoshino et al., 2018), others have also shown that high doses of NR in mice can be detrimental for mouse metabolic physiology (Shi et al., 2017), which can possibly be explained by differences in genetic backgrounds or composition of the diets.

High levels of NADH, the reduced form of vitamin B3, drive electron flow through the ETS, while high levels of ATP and low levels of oxygen impede electron flow. Recently it was shown that acute physiological hypoxia increases NADH levels and induces ROS, especially in the intermembrane space (Arias-Mayenco et al., 2018). At the same time the activity of SDH was inhibited, resulting in increased levels of succinate (Arias-Mayenco et al., 2018). NADH levels also play a role during I/R, where increased NADH levels drive malate to fumarate conversion during ischemia, and the increased succinate levels at CII that are produced during reperfusion cause increased mitochondrial ROS production, which accounts for the oxidative damage and cell death during I/R (Chouchani et al., 2014). Although no studies have been performed that focus on the relation between NADH levels, mitochondrial ROS, succinate and nuclear pathways, it would be interesting to see how succinate communicates with nuclear dioxygenases upon increased mitochondrial ROS. Although this communication remains elusive, a recent study illustrated how vitamin B3 metabolism, anti-oxidant responses and nuclear signaling are connected (Wei et al., 2017). Wei et al. (2017) showed that supplementation of NMN could alter nuclear signaling via modulation of Nrf2 expression and its translocation to the nucleus (Wei et al., 2017).

In addition to vitamin B2 and B3, vitamin B6 and B12 also play a pivotal role in redox homeostasis as they both operate in GSH metabolism (Figure 6). So far only a few studies have focused on the possible protective role of vitamin B6 (pyridoxine) or pyridoxine derivatives in amelioration of oxidative stress (Ren et al., 2016; Zhang et al., 2016; Roh et al., 2018). Pyridoxine increased antioxidant responses, possibly via upregulation of nuclear Nrf2 gene expression, and decreased levels of mitochondrial ROS (Ren et al., 2016). Pyridoxal-5′-phosphate supplementation resulted in reduced NLRP3 inflammasome activation and inflammatory cytokine production in peritoneal macrophages (Zhang et al., 2016), which couples mitochondrial-derived ROS production to nuclear signaling pathways in immune cells. However, Zhang et al. (2016) also pointed out that the outcomes for different pyridoxine derivatives are not always similar (Zhang et al., 2016), indicating that more research is needed to elucidate the mechanisms by which pyridoxine potentially prevents ROS-induced oxidative stress, and how these signals are possibly transferred to the nucleus.

Methylcobalamin (vitamin B12) contributes to GSH synthesis by acting as coenzyme for MS. The lack of methylcobalamin that is observed upon cobalamin deficiency is therefore associated with dysregulated GSH metabolism and increased ROS levels (Figure 6) (Pastore et al., 2014), and cobalamin deficiency is clinically characterized by reduced plasma levels of GSH and impaired anti-oxidant capacity (Misra et al., 2017). Of note, although adenosylcobalamin is not directly involved in the GSH cycle, a lack of adenosylcobalamin that is observed during MUT dysfunction is also linked to impaired GSH metabolism and increased ROS levels as it hampers mitochondrial function (Chandler et al., 2009; Richard et al., 2009).

Although vitamin B1 (thiamine) and vitamin B11 (folate) do not directly assist the ETS or GSH metabolism, both B-vitamins can alter cellular redox states. Thiamine can influence mitochondrial ROS production by acting as a cofactor for PDH and OGDH, two other major sources of mitochondrial superoxide (Figure 6). Thiamine deficiency is therefore linked to increased mitochondrial ROS formation (Bunik and Fernie, 2009; Ambrus et al., 2015). Since CIII-derived mitochondrial ROS can alter HIF1 signaling (Hamanaka et al., 2016), the higher levels of ROS that are observed in thiamine deficiency have perhaps also a signaling role toward HIF1. This would also partly explain why thiamine deficiency is associated with HIF1A stabilization (Zera and Zastre, 2018). Another interesting link between vitamin B1 and redox state was discovered recently (Tapias et al., 2018). Benfotiamine, a synthetic *S*-acyl derivative of thiamine, was found to induce the expression of nrf2 and ARE-dependent genes (Tapias et al., 2018). It even restored mitochondrial function in a mouse model of Alzheimer’s disease, which indicates that this thiamine derivative can link vitamin B1 metabolism and mitochondrial function to nuclear anti-oxidant defense systems (Tapias et al., 2018). Furthermore, it has also been shown that folate metabolism is linked to cellular redox states, in addition to its predominant role in supporting nucleic acid synthesis (Fan et al., 2014). Using quantitative flux analyses, the authors observed that oxidation of methylene tetrahydrofolate to 10-formyl-tetrahydrofolate was coupled to the reduction of NADP^+^ to NADPH, which showed that folate metabolism is essential to create reducing power in the cell (Fan et al., 2014). They also demonstrated that depletion of cytosolic as well as mitochondrial MTHFD resulted in lower NADPH/NADP^+^ and GSH/GSSG ratios with concomitant increased sensitivity to oxidative stress. These results showed that folate metabolism contributes to cellular redox states (Fan et al., 2014).

Recently, it was also identified that vitamin B5 (CoA) has an important role in redox regulation by functioning as a protective thiol (Tsuchiya et al., 2017, 2018). CoA was covalently linked to cellular proteins of mammalian cells in response to oxidizing agents and metabolic stress and induced a reversible PTM that was identified as protein CoAlation (Figure 6) (Tsuchiya et al., 2017, 2018). Proteins involved in redox homeostasis are particularly sensitive for CoAlation as they exhibit reactive thiol residues that can form homo- and heterodisulphides with CoA, such as GSH (Luo et al., 2006). Therefore, CoA has now also been implicated as an important regulator of redox homeostasis and could possibly also have nuclear targets.

## Conclusion

Overall, B-vitamins are of critical importance for regulating mitochondria, mitochondrial metabolites and signaling of mitochondrial metabolites to the nucleus. Although B-vitamins are among the oldest studied molecules in relation to health and disease, the revival of studying metabolism in pathologies, which were previously less well understood to be of metabolic origin, like cancer and immunological diseases, makes the B-vitamins highly relevant to be studied in light of novel therapeutic target development. In addition, the role of B-vitamins as essential dietary components makes it important to understand their nutritional role in relation to mito-nuclear signaling. All together this could serve as a proxy for understanding healthy dietary life styles and healthy aging.

## Author Contributions

JJ reviewed the literature and prepared the first manuscript. SG, JK, and VdB reviewed the literature, participated in writing and editing of the manuscript. VdB supervised the project and compiled the final version of the manuscript.

## Conflict of Interest Statement

The authors declare that the research was conducted in the absence of any commercial or financial relationships that could be construed as a potential conflict of interest.
